# Near Infrared Fluorescence Imaging of Intraperitoneal Ovarian Tumors in Mice Using Erythrocyte-Derived Optical Nanoparticles and Spatially-Modulated Illumination

**DOI:** 10.3390/cancers13112544

**Published:** 2021-05-22

**Authors:** Joshua M. Burns, Elise Shafer, Raviraj Vankayala, Vikas Kundra, Bahman Anvari

**Affiliations:** 1 Department of Bioengineering, University of California, 900 University Ave., Riverside, CA 92521, USA; jburn006@ucr.edu (J.M.B.); escha006@ucr.edu (E.S.); rvankayala@iitj.ac.in (R.V.); 2 Radoptics, LLC, 1002 Health Science Rd. E., Suite P214, Irvine, CA 92612, USA; 3 Department of Cancer Systems Imaging and Department of Radiology, The University of Texas MD Anderson Cancer Center, 1515 Holcombe Blvd, #57, Houston, TX 77030, USA; VKundra@mdanderson.org

**Keywords:** biomaterials, biomimetics, indocyanine green, nanomaterials, red blood cells

## Abstract

**Simple Summary:**

Ovarian cancer has a greater mortality rate than all gynecological malignancies combined. While cytoreductive surgery remains the primary therapeutic approach, its success is limited by the inability to visualize all tumor nodules for resection. We developed light activated nano-sized particles derived from red blood cells as potential imaging probes for near infrared fluorescence imaging of tumors during cytoreductive surgery. We present the first demonstration of the use of these nanoparticles in conjunction a spatially-modulated illumination (SMI) modality to image ovarian intraperitoneal tumors in mice. Our findings indicate that, at 24 h post-administration, these nanoparticles accumulated at higher levels in tumors as compared to organs, and that use of SMI enhances the image contrast.

**Abstract:**

Ovarian cancer is the deadliest gynecological cancer. Cytoreductive surgery to remove primary and intraperitoneal tumor deposits remains as the standard therapeutic approach. However, lack of an intraoperative image-guided approach to enable the visualization of all tumors can result in incomplete cytoreduction and recurrence. We engineered nano-sized particles derived from erythrocytes that encapsulate the near infrared (NIR) fluorochrome, indocyanine green, as potential imaging probes for tumor visualization during cytoreductive surgery. Herein, we present the first demonstration of the use of these nanoparticles in conjunction with spatially-modulated illumination (SMI), at spatial frequencies in the range of 0–0.5 mm^−1^, to fluorescently image intraperitoneal ovarian tumors in mice. Results of our animal studies suggest that the nanoparticles accumulated at higher levels within tumors 24 h post-intraperitoneal injection as compared to various other organs. We demonstrate that, under the imaging specifications reported here, use of these nanoparticles in conjunction with SMI enhances the fluorescence image contrast between intraperitoneal tumors and liver, and between intraperitoneal tumors and spleen by nearly 2.1, and 3.0 times, respectively, at the spatial frequency of 0.2 mm^−1^ as compared to the contrast values at spatially-uniform (non-modulated) illumination. These results suggest that the combination of erythrocyte-derived NIR nanoparticles and structured illumination provides a promising approach for intraoperative fluorescence imaging of ovarian tumor nodules at enhanced contrast.

## 1. Introduction

Ovarian cancer has the greatest mortality rate than all gynecological malignancies combined [1]. Epithelial ovarian cancer (EOC) arising from the ovarian surface epithelium accounts for nearly 85% of ovarian tumors [2]. EOCs have relatively shallow invasion depths limited to less than 5 mm [3]. The majority of patients with ovarian cancer are often diagnosed with the late stage disease when the cancer has become peritoneal, spreading outside the pelvis and to other parts of the abdomen, onto the omentum, and surface of organs such as the liver or spleen [4]. The 5-year survival rate is relatively high (~92%) if diagnosed at stage I when the cancer is still localized in one or both ovaries or the fallopian tubes, but significantly drops to less than 30% if diagnosed at stage III-IV where the cancer metastasizes beyond the pelvic region [4].

One of the most important factors for improving survival rate is the success of cytoreductive surgery with complete resection of all visible cancer [5,6,7]. Several studies recommend resecting all “visible” lesions, and not just cytoreduction of tumor nodules <1 cm as the surgical goal [8,9]. However, a current problem with cytoreductive surgery is lack of an intraoperative image-guided approach to enable the surgeon to visualize all tumor nodules for resection, as well as the poor contrast between the tumor and healthy tissue as the lesions are whitish/pink and often appear similar to normal tissues. Therefore, there is a clinical need for development of intraoperative imaging systems to guide the removal of all intraperitoneal ovarian tumors for complete resection. For example, the effectiveness of fluorescein isothiocyanate, a visible fluorescent tracer, for intraoperative image-guided removal of ovarian tumors has been demonstrated [10].

Use of near infrared (NIR) wavelengths (~700–2500 nm) are particularly advantageous since there is minimal autofluorescence by tissues over this spectral band. As a result, improved image contrast can be achieved by use of exogenous NIR fluorescent dyes. To date, Indocyanine green (ICG) remains the only FDA-approved NIR fluorochrome for specific clinical applications including assessment of cardiac and hepatic function, ophthalmic angiography, and blood flow evaluation [11,12,13]. ICG has also been used during intraoperative imaging of liver and ovarian cancers [14,15]. However, one of the major drawbacks of ICG is its short circulation half-life (~3–5 min) [16,17] that results from its binding to plasma proteins (mainly albumin) and lipoproteins, with subsequent and elimination from the body through the hepatobiliary mechanism.

Various constructs (e.g., micelles, liposomes, synthetic polymers) have been used to overcome these limitations by encapsulating ICG [18,19,20,21,22]. Due to their potential biocompatibility, erythrocytes, macrophages and lymphocytes or constructs derived from them have been receiving attention as delivery platforms [23,24,25]. Erythrocytes are particularly attractive due to the presence of “self-marker” membrane proteins, including CD47, that shield them from phagocytosis by macrophages [26,27]. Therefore, constructs derived from appropriately engineered red blood cells (RBCs) may have extended circulation times to have their cargo (e.g., ICG) available for an intended application such as optical imaging of ovarian tumor nodules before removal by macrophages. We demonstrated the first engineering of nano-sized particles derived from erythrocytes containing ICG [28]. For brevity, we refer to these particles as nRBCs-ICG (nanoparticles derived from RBCs and doped with ICG). Once activated by NIR light, these particles can transduce the energy to emit fluorescence, generate heat, or lead to formation of reactive oxygen species [29,30]. When combined with appropriate imaging techniques, nRBCs-ICG can provide detection of specific structures such as tumors.

A particular optical imaging method is based on spatially-modulated illumination (SMI). Using this method, structured illumination patterns of varying spatial frequencies are projected on the tissue, and the attenuated intensity of the spatial patterns returning from the illuminated object is measured by optical sensors on a pixel-by-pixel basis. SMI has been used to estimate the 2-D optical properties of tissues [31,32], and quantify depth-resolved Protoporphyrin IX concentrations from preclinical glioma models [33,34]. In addition, SMI has been used in conjunction with the far-red fluorochrome, Cy5, for enhanced fluorescence imaging of subcutaneous myeloma tumor boundaries [35].

Herein, we present the first demonstration of the use of nRBCs-ICG in conjunction with SMI to fluorescently image intraperitoneal ovarian tumors in mice. Specifically, in this preliminary study, we hypothesize that SMI can enhance the fluorescence image contrast between intraperitoneal tumors and peritoneal organs when used in combination with nRBCs-ICG. We find that by using nRBCs-ICG and SMI, the fluorescence image contract is enhanced by at least two-fold compared to spatially-uniform (DC) illumination. Our results suggest that the combination of nRBCs-ICG and structured illumination may provide a promising approach for fluorescence imaging of ovarian intraperitoneal tumors.

## 2. Materials and Methods

### 2.1. Fabrication of nRBCs-ICG

We centrifuged 10 mL of whole human blood (Innovative Research, Novi, MI, USA) for 5 min at 1300× *g* and 4 °C to isolate the erythrocytes. Plasma and buffy coat were discarded and the remaining pellet containing erythrocytes was washed twice with 310 mOsm phosphate buffer saline (PBS) (referred to as the 1× solution) (pH~8.0). The erythrocytes were then subjected to hypotonic treatment using 0.25× PBS (77.5 mOsm, pH~8.0) and centrifuged (20,000× *g*, 15 min, 4 °C). This process was repeated until all the hemoglobin was depleted, resulting in micro-sized erythrocyte ghosts (µEGs).

Nano-sized erythrocyte ghosts (nEGs) were produced by sequential extrusion of µEGs through 800, 400, and 200 nm polycarbonate porous membranes (Nuclepore Track-Etched Membranes, Whatman, Florham Park, New York, NY, USA) using an extruder (LIPEX Extruder, TRANSFERRA Nanosciences Inc, Burnaby, Canada) with the extrusion process repeated 10 times through each of the membranes with the indicated pore diameters. To concentrate the nano-sized EGs, 10 mL of suspension was centrifuged (99,000× *g*, 1 h, 4 °C), and the pellet was then re-suspended in 2 mL of 1× PBS.

To load ICG into nEGs and form nRBCs-ICG, 2 mL of the concentrated nEGs suspension was incubated with 2 mL of 75 μM ICG dissolved in water and 2 mL of hypotonic buffer (Na_2_HPO_4_/NaH_2_PO_4_, 140 mOsm, pH~8.0), resulting in a 25 μM concentration of ICG in the loading buffer. The suspension was incubated for five minutes at 4 °C in the dark, centrifuged at 74,000× *g* for 30 min, and then washed two times using 1× PBS to remove any non-encapsulated ICG. The pellet containing ICG-encapsulated nanoparticles (i.e., nRBCs-ICG) was removed and re-suspended in 0.5 mL of 1× PBS (4 °C). To avoid saturation in the recordings of the optical spectra, this solution of nRBCs-ICG was further diluted by a factor of 200 using 1× PBS. We then acquired the optical density (OD) and fluorescence spectra of the 1:200 diluted solution of nRBCs-ICG.

We estimated the loading efficiency of ICG into the nanoparticles as follows. We first acquired the absorption spectra of free ICG dissolved in water in concentration range of 2−10 μM. ICG absorption in water varies linearly for concentrations less than about 15 µM [36,37]. We chose water as the solvent since the absorption spectra of the nanoparticles resembles that of ICG dissolved in water at concentrations less than ~20 μM. The spectra were then spectrally integrated in the range of 600−900 nm, and the resulting values (*A*_int_) were fitted against ICG concentrations to obtain a calibration curve (R^2^ = 0.99). We then used the value of *A*_int_ associated with the supernatant solution, obtained at the end of the fabrication process, in conjunction with the calibration curve to estimate the amount of ICG remaining within the supernatant (~2 µg). Based on the initial amount of ICG introduced in the loading buffer (116.25 µg), the estimated loading efficiency was ~98%.

### 2.2. Size and Optical Characterization of nRBCs-ICG

The hydrodynamic diameters of nRBCs-ICG suspended in 1× PBS were estimated by dynamic light scattering (DLS) (Zetasizer NanoZS90, Malvern Instruments Ltd., Malvern, UK). OD of the nRBCs-ICG suspension (diluted 1:200) in 1× PBS was obtained using a UV-visible spectrophotometer (Cary 50 UV-Vis spectrophotometer, Agilent Technologies, Santa Clara, CA, USA) with an optical path length of 1 cm. The fluorescence emission spectra of nRBCs-ICG (diluted 1:200) was acquired in response to 710 ± 2.5 nm and recorded using a fluorimeter (Fluorolog-3 spectrofluorometer, Edison, NJ, USA) for emission wavelengths greater than 725 nm. We normalized the fluorescence emission spectra *χ*(*λ*) as:

(1) $$\chi (\lambda )=\frac{F(\lambda )}{\left(1-10^{-OD(\lambda_{ex})}\right)}$$

where *F* is the wavelength (*λ*)-dependent intensity of the emitted fluorescence light, and *λ*_ex_ is the excitation wavelength. We have previously reported comprehensive studies related to characterization of nRBCs-ICG and their physical and optical stability [38,39]. Therefore, such characterizations are not repeated here as they are not the subject of this study. We have also previously published transmission and scanning electron microscopic images of nRBCs-ICG [28,40,41].

### 2.3. SMI System

**Figure 1:**
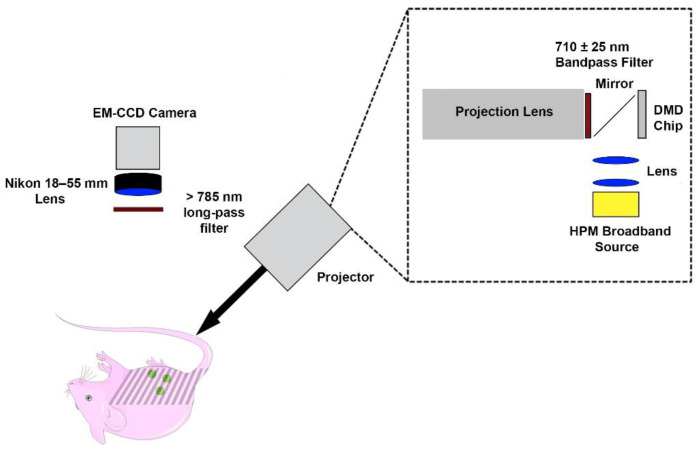
Spatially-modulated illumination system for fluorescence imaging of intraperitoneal ovarian tumors.

The SMI imaging system consisted of a digital micromirror device (DMD) chip projector equipped with a broadband high-pressure mercury (HPM) lamp (Dell MP2400) (Figure 1). Illumination light was optically filtered to deliver excitation in the range of 710 ± 25 nm. An electron multiplier gained charge coupled device camera (Quant EM-CCD, C9100-14 Hamamatsu) equipped with an 18–55 mm focal length lens (Nikon AF-S DX NIKKOR 18–55 mm), and a long-pass filter (>785 nm) was used to collect fluorescence emission. Exposure times of the camera were set to 2 s for fluorescence imaging.

We created patterned grayscale sinusoidal illumination with spatial intensity pattern (*S*):

(2) $$S=\frac{S_{o}[1+\cos(2\pi f_{x}x+\theta )]}{2}$$

where *S*_o_, *f*_x_ and θ are the illumination source intensity, spatial modulation frequency, and spatial phase offset, respectively. We used uniformly spaced values of *f*_x_ ranging between 0 and 0.50 mm^−1^ and θ = 0, 120 and 240°. For each *f*_x_, we averaged three images at a given phase, and obtained the amplitude envelope (*M*_AC_) by using a three-point amplitude demodulation method [31]:

(3) $$M_{\mathrm{AC}}(x,y)=\frac{\sqrt{2\{{\left[I_{1}(x,y)-I_{2}(x,y)\right]}^{2}+{\left[I_{2}(x,y)-I_{3}(x,y)\right]}^{2}+{\left[I_{3}(x,y)-I_{1}(x,y)\right]}^{2}\}}}{3}$$

where *I*_1_, *I*_2_ and *I*_3_ represent the average intensity values of three images at a pixel in a given *x* and *y* position and a particular phase. We averaged three images to determine the amplitude of the DC (*f*_x_ = 0 mm^−1^) component:

(4) $$M_{\mathrm{DC}}(x,y)=\frac{I_{1}(x,y)+I_{2}(x,y)+I_{3}(x,y)}{3}$$

### 2.4. Animal Study

We used five female Nu/J mice (20~25 g; 6–8 weeks) (Jackson Laboratory, Bar Harbor, Maine) under a protocol approved by the University of California, Riverside Institutional Animal Care and Use Committee (A-20170038). We implanted ~1 × 10^7^ SKOV3 cancer cells by intraperitoneal (IP) injection while each animal was anesthetized by 2% isoflurane in oxygen. After four weeks, we administered 100 µL of nRBCs-ICG suspension in 1× PBS by IP injection while the animals were anesthetized by 2% isoflurane in oxygen.

At 24 h post injection of nRBCs-ICG, mice were anesthetized with ketamine (80 mg/kg)/xylazine (10 mg/kg). We then acquired color photographs and SMI-based images of the whole body with uniformly spaced *f*_x_ ranging between 0 and 0.50 mm^−1^. Mice were then euthanized with compressed CO_2_ gas, and the abdomen was opened to expose the organs. We washed to abdominal cavity three times with 1× PBS to remove nRBCs-ICG that were not taken up by tumors and organs. The open abdomen was then imaged by the SMI system with uniformly spaced *f*_x_ ranging between 0 and 0.50 mm^−1^. For all representative images, fluorescence emission intensities were normalized by dividing the values by the maximum intensity.

Following euthanasia, liver, spleen, lungs, intestine, heart, kidney and tumors with surrounding tissue were extracted and fluorescently imaged by the SMI system. Regions of interest (ROIs) were selected for each organ, and the mean intensity (*Ī*) was calculated as:

(5) $$\bar{I}=\frac{\sum_{j=1}^{m}I_{j}}{m}$$

where *m* is the total number of pixels in the ROI and *I_j_* is the pixel intensity at the *j*th pixel of a given image.

We subsequently used the estimated *Ī* values to compute the contrast between the tumor and a given organ as:

(6) $$Contrast=\frac{{\bar{I}}_{T}-{\bar{I}}_{O}}{{\bar{I}}_{O}}$$

where *Ī_T_* and *Ī_O_* represent the respective *Ī* values for the tumor and each organ (liver, spleen, lungs, intestine, heart, kidney).

### 2.5. Statistical Analysis

Using the Shapiro–Wilk test, we validated that the computed normalized intensities and contrast values had normal distributions. We used a one-tail paired *t*-test to compare the normalized intensity values at *f*_x_ = 0.1 mm^−1^ with those obtained using *f*_x_ = 0.5 mm^−1^ for tumors and all organs. We used a one-way repeated measures analysis of variance (ANOVA) to analyze the contrast between tumor and liver for *f*_x_ = 0.1–0.5 mm^−1^ with the contrast at *f*_x_ = 0. Following the ANOVA, we used a one-tail paired *t*-test to compare the contrast values between tumors and all organs at *f*_x_ = 0.2 mm^−1^ with those obtained at *f*_x_ = 0 mm^−1^.

## 3. Results and Discussion

### 3.1. Size and Optical Characteristics of nRBCs-ICG

**Figure 2:**
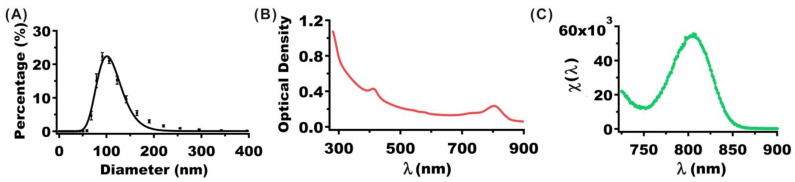
(**A**) Hydrodynamic diameter distribution of nRBCs-ICG as determined by DLS. Squares and error bars represent the measured mean and standard deviation (SD) of diameters, respectively (*n* = 8 measurements of the same sample). The estimated mean peak diameter as determined from a log-normal fit (solid curves) was ~101 nm. (**B**) OD spectrum of nRBCs-ICG. (**C**) Normalized fluorescence spectrum (see Equation (1)) of nRBCs-ICG in response to photo-excitation at 710 nm. The suspension of nRBCs-ICG in 1× PBS was diluted by factor of 200 for both OD and Fluorescence measurements. ICG concentration used in the loading buffer to fabricate these nRBCs-ICG was 25 µM.

Illustrative hydrodynamic diameter distribution, OD, and fluorescence spectra of nRBCs-ICG fabricated using 25 µM ICG in the loading buffer are presented in Figure 2. The estimated mean peak diameter of nRBCs-ICG as determined by fitting a log-normal curve to the measured values by dynamic light scattering was ~101 nm (Figure 2A). These results are consistent with the measurements of the nRBCs-ICG diameter by transmission and electron scanning imaging [28,40,41]. Since the hydrodynamic diameters of nRBCs-ICG are <200 nm, they are likely to be effective for extravasation into tumors through the enhanced permeability and retention (EPR) effect, induced by the leaky tumor vasculature and impaired lymphatic drainage [42,43].

The OD spectrum (Figure 2B) is a measure of the wavelength-dependent intensity of the transmitted light after absorption and scattering of light by nRBCs-ICG. The OD value at 280 nm includes a contribution from the absorbed light by the proteins in nRBCs-ICG. OD values in the range of 600–900 nm with spectral peak and shoulder at 805 and 755 nm, respectively, include contributions from absorbed light by monomeric and aggregated forms of ICG [28,38]. Fluorescence spectrum in response to photo-excitation at 710 nm demonstrated an emission peak at 804 nm corresponding to the monomeric form of ICG (Figure 2C).

We recently reported the results of an extensive study related to the physicochemical properties of RBC-derived particles doped with ICG as function of the size of the particles at both nano and micro scales [38]. These properties included ICG concentration-dependent zeta-potentials, absorption and emission spectra, excitation-emission maps, and relative fluorescence quantum yield. For example, the zeta-potential of nRBCs-ICG was ~−12.5 mV, and not significantly different from that for RBCs (−12.8 mV), indicating that the carboxyl groups of sialoglycoproteins, which are associated with much of the negative charge of RBCs, were retained during the fabrication of the particles. After 12 h of storage in the dark at 4 and 37 °C, fluorescence emission of nRBCs-ICG was retained, whereas there was nearly 40% reduction in the emission for free ICG [38]. We determined that only about 5% of ICG leaks from nRBCs-ICG over 48 h of storage at 37 °C [41]. We also found that there is only about a 5% reduction in ICG monomer absorbance of ICG after 8 days of storage in isotonic PBS at 4°C in the dark [40].

We have previously investigated the optical and physical properties of nRBCs-ICG stored at −20 °C for up to 8 weeks and then thawed at room temperature [39]. Our results showed that the hydrodynamic diameter, zeta-potential, absorbance, and NIR fluorescence emission of nRBCs-ICG were retained following the freeze–thaw cycle. The ability of nRBCs-ICG in NIR fluorescence imaging of ovarian cancer cells, as well as their biodistribution in reticuloendothelial organs of healthy Swiss Webster (SW) mice after the freeze–thaw cycle were similar to those for freshly prepared nRBCs-ICG.

### 3.2. Animal Imaging

**Figure 3:**
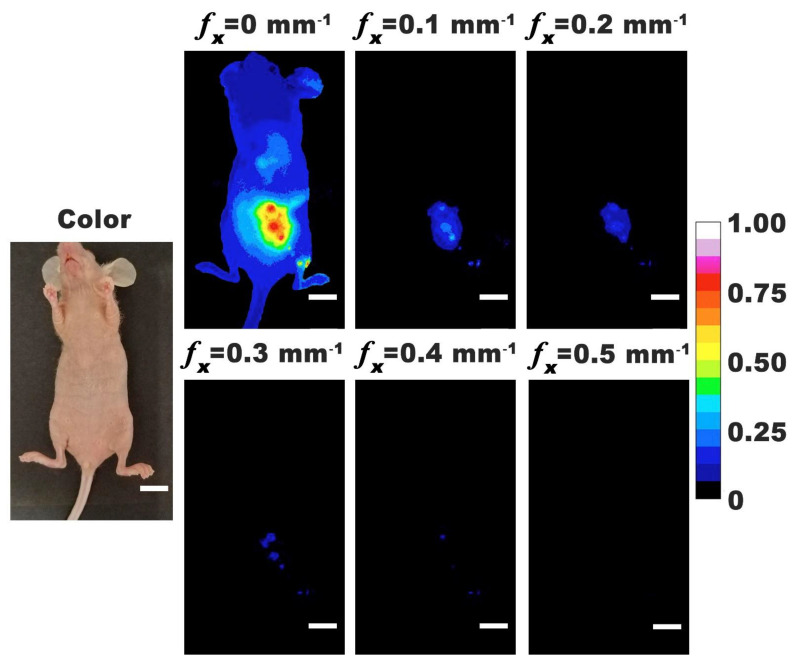
Representative color and normalized fluorescence images for a closed-body mouse with ovarian intraperitoneal tumors. Images were obtained at 24 h post-intraperitoneal injection of nRBCs-ICG. SMI-based imaging at spatial frequencies in the range of 0–0.5 mm^−1^ are presented. The color scale bar corresponds to normalized intensity associated with the fluorescent images. White scale bars on images correspond to 10 mm.

We show representative photographic color and normalized fluorescence images for a closed body mouse (Figure 3). NIR emission was most intense under DC (non-structured) illumination (*f*_x_ = 0) with progressively lower signal levels as *f*_x_ was increased from 0.1 to 0.5 mm^−1^. These results indicate that nRBCs-ICG were present in the abdominal cavity at 24 h post IP injection, and emitted NIR fluorescence that could still be detected despite attenuation of the emitted light by skin and other structures.

**Figure 4:**
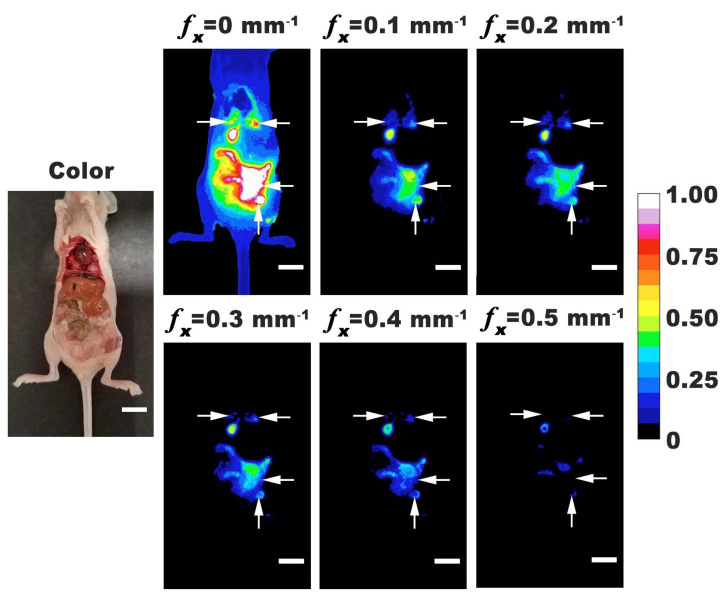
Representative color and normalized fluorescence images for an open-body mouse with ovarian intraperitoneal tumors. Images were obtained at 24 h post-intraperitoneal injection of nRBCs-ICG. SMI-based imaging at spatial frequencies in the range of 0–0.5 mm^−1^ are presented. The color scale bar corresponds to normalized intensity associated with the fluorescent images. Arrows indicate tumor locations. White scale bars on images correspond to 10 mm.

To validate the co-registration of color and SMI-based NIR fluorescence images, mice were imaged under opened abdomen. Representative color and normalized fluorescence intensity images with *f*_x_ = 0–0.50 mm^−1^ are shown in Figure 4. For all mice, there were tumor nodules identified throughout the abdominal cavity. In some mice, tumors could be identified in thoracic cavity near the lungs and heart. These results indicate that tumors metastasized above the diaphragm and could be identified by nRBCs-ICG. Localization of nRBCs-ICG at these distant metastatic sites can result from their re-entry into the systemic circulation through the portal vein since the visceral peritoneum, mesentery, and omentum drain into the portal system, or through the parietal peritoneum that drains into the lymphatic system [44]. Given the larger surface area of the membranes draining into the portal vein, and the slow rate of lymphatic flow [44], nRBCs-ICG are likely to more quickly enter into the circulation through the portal vein. While the greatest emission intensity was observed under DC illumination, tumors could still be visualized by SMI-based imaging with *f*_x_ in the range of 0.1 to 0.5 mm^−1^. A possible mechanism for accumulation of nRBCs-ICG in tumors is via the EPR effect, after the particles gain entry into the circulation. Following IP administration, some particles may also reach the tumors by convective and diffusive transport mechanisms.

**Figure 5:**
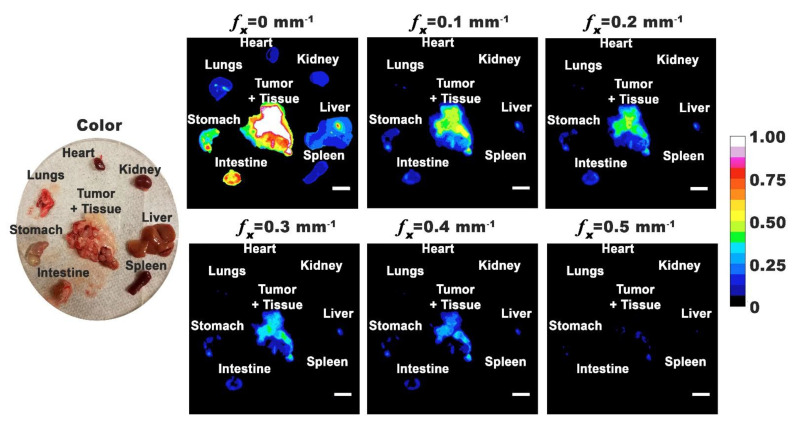
Representative color and normalized fluorescence images for excised organs from a mouse with ovarian intraperitoneal tumors. Images were obtained at 24 h post-intraperitoneal injection of nRBCs-ICG. SMI-based imaging at spatial frequencies in the range of 0–0.5 mm^−1^ are presented. The color scale bar corresponds to normalized intensity associated with the fluorescent images. White scale bars on images correspond to 10 mm.

We extracted the tumors and various organs from the body and fluorescently imaged them using SMI. Representative excised organs are shown in Figure 5. Due to the difficulty in visually distinguishing the tumor margins around healthy tissue, we removed the surrounding tissues. In addition to tumor masses growing independently, there were also tumor masses attached to the organs. Most intense emission intensities were associated with excised tumor and specific organs (liver, intestine, and stomach) under DC illumination. However, when using SMI, the emission intensities from tumor were higher as compared to those from organs, suggesting that higher amounts of nRBCs-ICG were present within the tumor.

Emission from the liver and intestine is consistent with the hepatobiliary elimination mechanism, which is also the main elimination pathway for ICG. In accordance with this mechanism, once into the liver sinusoids, some fraction of nRBCs-ICG can leave the sinusoids through the pores between the fenestrated lining of the endothelial cells [45,46], and extravasate into the space of Disse where they can go undergo endocytosis by hepatocytes and, subsequently, be secreted into the bile ducts, passage into duodenum, and finally be eliminated from the body. In addition to the hepatobiliary elimination pathway, some of the extravasated nRBCs-ICG may be taken up by hepatic stellate cells located within the space of Disse. The fraction of nRBCs-ICG that are not extravasated out of the sinusoids may undergo phagocytosis by the Kupffer macrophages adherent to the endothelial lining of the liver sinusoids. Fluorescence emission from the stomach could be due to the presence of tumors within the organ, as well as chlorophyll (alfalfa) in the rodent diet [47].

In turbid media, subject to SMI, the effective attenuation coefficient (*µ*′*_eff_*) of the inbound excitation light as well as the outbound fluorescence light is given as [31,48]:

(7) $${\mu^{\prime }}_{eff}=\sqrt{\mu_{eff}^{2}+{\left(2\pi f_{x}\right)}^{2}}=\sqrt{3\mu_{a}(\mu_{a}+{\mu^{\prime }}_{s})+{\left(2\pi f_{x}\right)}^{2}}$$

where *µ*_a_ and *µ*_s_’ are the respective absorption and reduced coefficients of the tissue. The parameter *µ*_s_’ is related to the scattering coefficient (*µ*_s_) as *µ*_s_’= *µ*_s_ (1 − *g*) where *g* is the anisotropy factor associated with scattering, and ~0.95 for biological tissues [49]. At *f*_x_ = 0, the intensity of both inbound and outbound light will be attenuated according to the endogenous optical properties of the tumor (or the organs) as well as those of the nRBCs-ICG. Upon spatial modulation of the excitation light, there is an additional component (2π*f*_x_) to the attenuation of the inbound and outbound light, which increases with the applied spatial frequency.

Fluorescence intensity measured at the surface (*F_surface_*) is a spatially integrated emission that originates approximately at depth of *δ′_eff_* = (1/*µ*^’^*_eff_*) and encompasses the emission from the overlying layers:

(8) $$F_{surface}=\int_{{\delta^{\prime }}_{eff}}^{0}F(z)e^{-{\mu^{\prime }}_{eff}z}dz$$

**Figure 6:**
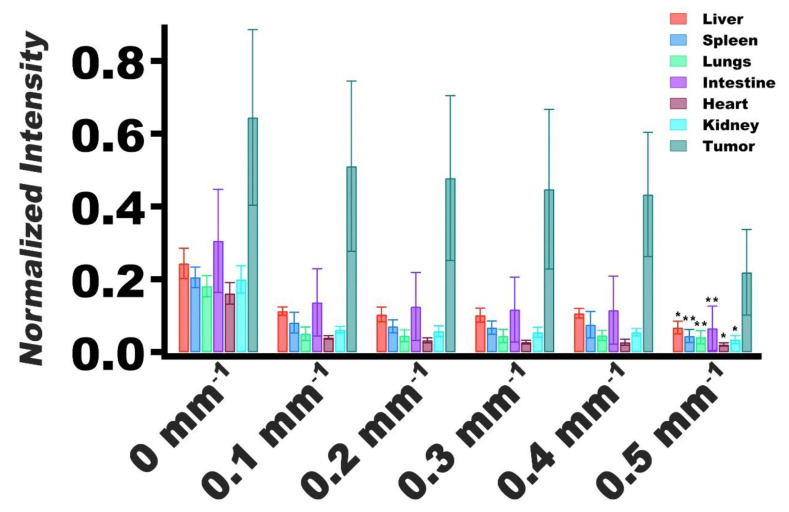
Normalized values of fluorescence emission intensity mean and SDs (error bars) of extracted tumors and organs (*n* = 5 for each organ and tumors) for spatial frequencies of 0–0.5 mm^−1^. Emission intensities were normalized by dividing the average intensity of the extracted organ or tumor by the maximum intensity of the image. Statistically significant differences in mean normalized intensity values between imaging at *f*_x_ = 0.5 mm^−1^ and *f*_x_ = 0.1 mm^−1^ are shown by asterisks with * indicating *p* < 0.05, and ** indicating *p* < 0.01.

Upon changing *f*_x_ from 0 mm^−1^ (DC) to the lowest spatial modulation frequency (0.1 mm^−1^), there was a decline in normalized fluorescence emission intensity values for the tumor and various organs (Figure 6). In particular, there were statistically significant differences in the mean normalized intensity values for all organs imaged at *f*_x_ = 0.5 mm^−1^ as compared to imaging at *f*_x_ = 0.1 mm^−1^. These results imply that the highest spatial frequency did not propagate as deeply into the organs, indicating that the organs acted as low-pass filters [32,48,50,51].

For tumors, the mean normalized intensity at *f*_x_ = 0.5 mm^−1^ was not significantly different from the mean value associated with imaging at *f*_x_ = 0.1 mm^−1^ (*p* = 0.062) (Figure 6). These results suggest that the effective optical properties of the tumors were distinct from the other organs, and that the tumors did not resemble the same low-pass filtering behavior as the organs. Furthermore, these results suggest that there was greater accumulation of nRBCs-ICG in the tumors as compared with the organs, evidenced by higher normalized intensity values associated with tumors.

The reported mean values of *µ*_a_ and *µ*_s_’ for malignant ovarian tissues at 730 nm are ~0.048 mm^−1^ and 0.040 mm^−1^ (assuming *g* ~0.95) [52]. Our previous results indicate that the values of *µ*_a_ and *µ*_s_’ for nRBCs-ICG at 710 nm are of the order of 0.5 and 0.1 mm^−1^, respectively [53]. For tumors containing nRBCs-ICG at a given fractional volume (*f*), the effective absorption coefficient (*µ*_a,eff_) and reduced scattering coefficient (*µ*_s_’) can be expressed as:

*µ*_a,eff_ = *f µ*_a,nRBCs-ICG_ + (1 − *f*) *µ*_a,tumor_ (9)

*µ*_s,eff_’ = *f µ*_s,nRBCs-ICG_’ + (1 − *f*) *µ*_s,tumor_’ (10)

For example, if *f* = 0.05, and using the values of the optical properties indicated above for ovarian tumors and nRBCs-ICG, the estimated value of *µ*’*_eff_* based on Equation (7) at DC illumination is ~0.155 mm^−1^, corresponding to *δ’_eff_* ~6.45 mm. For *f*_x_ = 0.1 mm^−1^, and 0.5 mm^−1^, the estimated respective values of *µ*’*_eff_* become 0.646 mm^−1^ and 3.145 mm^−1^, with corresponding *δ’_eff_* values of 1.54 mm and 0.32 mm. Therefore, depending on the choice of *f*_x_, various depths of nRBCs-ICG-containing tumors (~0.32–6.45 mm as the first order of approximation) can be probed, with the recorded fluorescence intensity at the surface being a function of the probed depth (Equation (7)). Additionally, since these analyses indicate that the depth of imaging by SMI in conjunction with nRBCs-ICG is of the order of a few mm, the method is potentially suitable to image epithelial diseases such as EOC and the associated tumor margins.

**Figure 7:**
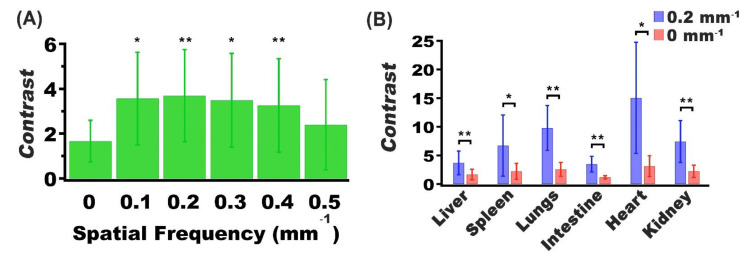
Mean and SD values of the contrast between (**A**) tumor and liver for spatial frequencies in the range of 0–0.5 mm^−1^, and (**B**) tumor and various organs for spatial frequencies of 0 and 0.2 mm^−1^. In both panels, *n* = 5 for each organ and tumor. In panel (**A**), asterisks indicate that the mean contrast value at those spatial frequency values (*f*_x_ = 0.1, 0.2, 0.3. and 0.4 mm^−1^) were signficantly different from the mean contast at DC imaging (*f*_x_ = 0.0). In panel (**B**), asterisks indicate statistically significant differences in mean contrast between imaging at *f*_x_ = 0.2, mm^−1^ and DC imaging for the indicated organs. In both panels, * and ** indicate *p* < 0.05, and *p* < 0.01, respectively.

Presence of nRBCs-ICG can also serve as the source of the optical contrast between the tumor and other organs. As an example, there was a significantly higher contrast in delineating the tumor from the liver for *f*_x_ = 0.10–0.40 mm^−1^ when compared to *f*_x_ = 0 mm^−1^ (Figure 7A). At *f*_x_ = 0.20 mm^−1^; there was greater contrast between tumors and other organs (Figure 7B). In particular, the image contrast values between the tumor and liver, and between the tumor and spleen, were enhanced by ~2.1 and 3.0 times, respectively, at *f*_x_ = 0.20 mm^−1^ as compared to the contrast values at DC illumination.

nRBCs-ICG are also amenable to surface functionalization with various biomolecules including folate, and antibodies (e.g., anti-HER2) [40,54,55]. Given the significance of molecular profiling for development of targeted therapeutics [56], nRBCs-ICG can be functionalized with personalized targeting moieties to enhance specificity towards cancerous ovarian cells.

We have previously reported on toxicological evaluation of nRBCs-ICG using blood chemistry and hematological profiling, and histological analysis [41]. Our results from that study showed that the levels of alanine aminotransferase and aspartate aminotransferase, enzymes associated with liver function, and the levels of creatinine and urea nitrogen, biomolecules associated with kidney function were not altered in a statistically significant manner at 24 h post injection of the particles in healthy SW mice. Values of RBC count, mean corpuscular volume, hemoglobin, and % hematocrit were not altered in response to administration of the particles. Similarly, histological sections of liver, spleen, lung, heart, and kidney did not show any pathological alterations.

In the animal models reported here, tumor diameters ranged between ~1 and 10 mm, but most of them had diameters between 2 and 3 mm. To determine the capability of SMI-based imaging in conjunction with these RBC-derived nanoparticles in detecting the smallest tumor nodules, longitudinal studies following implantation of the tumor cells are needed. Specifically, tumors will need to be imaged at various time points following implantation to determine if detection of tumor nodules < 1 mm is possible using this approach. Future studies will also include the optimization of the number concentration of nRBCs-ICG and the imaging time point as methods to further enhance the image contrast.

## 4. Conclusions

We presented the first demonstration of the use of nRBCs-ICG in conjunction with spatially modulated illumination for fluorescence imaging of intraperitoneal ovarian tumors in mice. Our results suggest that, at 24 h post intraperitoneal injection of nRBCs-ICG, higher quantities of these particles are accumulated within ovarian intraperitoneal tumor implants in mice compared to various other organs. We demonstrate that use of nRBCs-ICG in conjunction with spatially-modulated illumination enhances the image contrast as compared to DC illumination. These findings suggest that the combination of nRBCs-ICG and structured illumination provides a promising approach for fluorescence imaging of ovarian intraperitoneal tumors at improved contrast.

## Data Availability

Not applicable.
